# Volumetric modulated arc therapy with flattening filter free beams for isolated abdominal/pelvic lymph nodes: report of dosimetric and early clinical results in oligometastatic patients

**DOI:** 10.1186/1748-717X-7-204

**Published:** 2012-12-05

**Authors:** Filippo Alongi, Antonella Fogliata, Elena Clerici, Pierina Navarria, Angelo Tozzi, Tiziana Comito, Anna Maria Ascolese, Alessandro Clivio, Francesca Lobefalo, Giacomo Reggiori, Luca Cozzi, Pietro Mancosu, Stefano Tomatis, Marta Scorsetti

**Affiliations:** 1 IRCCS Istituto Clinico Humanitas, Radiation Oncology Dept, Rozzano-Milan, Italy; 2 Oncology Institute of Southern Switzerland, Medical Physics Unit, Bellinzona, Switzerland

**Keywords:** Abdominal/pelvic lymphnodes, VMAT, FFF

## Abstract

**Background:**

SBRT is a safe and efficient strategy to locally control multiple metastatic sites. While research in the physics domain for Flattening Filter Free Beams (FFF) beams is increasing, there are few clinical data of FFF beams in clinical practice. Here we reported dosimentric and early clinical data of SBRT and FFF delivery in isolated lymph node oligometastatic patients.

**Methods:**

Between October 2010 and March 2012, 34 patients were treated with SBRT for oligometastatic lymph node metastasis on a Varian TrueBeam^TM^ treatment machine using Volumetric Modulated Arc Therapy (RapidArc). We retrospectively evaluated a total of 25 patients for isolated lymph node metastases in abdomen and/or pelvis treated with SBRT and FFF (28 treatments). Acute toxicity was recorded. Local control evaluation was scored by means of CT scan and/or PET scan.

**Results:**

All dosimetric results are in line with what published for the same type of stereotactic abdominal lymph node metastases treatments and fractionation, using RapidArc. All 25 FFF SBRT patients completed the treatment. Acute gastrointestinal toxicity was minimal: one patient showed Grade 1 gastrointestinal toxicity. Three other patients presented Grade 2 toxicity. No Grade 3 or higher was recorded. All toxicities were recovered within one week. The preliminary clinical results at the median follow up of 195 days are: complete response in 12 cases, partial response in 11, stable disease in 5, with an overall response rate of 82%; no local progression was recorded.

**Conclusions:**

Data of dosimetrical findings and acute toxicity are excellent for patients treated with SBRT with VMAT using FFF beams. Preliminary clinical results showed a high rate of local control in irradiated lesion. Further data and longer follow up are needed to assess late toxicity and definitive clinical outcomes.

## Background

The detection of single or limited organ metastases, defined oligometastases, has been recently increased by advancements in imaging technology [1]. In this subgroup of cancer patients, local treatments for oligometastases have been widely investigated for many cancers with the objective to impact on disease control and survival [2]. In selected oligometastatic patients, surgical resection of limited metastatic burden of disease prolongs survival [1,2]. However, the ideal candidates for local therapy are difficult to identify.

In this scenario, radiation treatments can play a role to achieve local control of oligometastatic focal disease. As smaller foci of metastases are defined, highly conformal radiation therapy, such as Stereotactic Body Radiotherapy (SBRT) or similar techniques, can prove to be less invasive and more effective than surgery because of decreasing morbidity, less costs and the potential of delivering ablative doses on an outpatient basis. Emerging data show that SBRT, in its various treatment modalities, is a safe and efficient strategy to locally control multiple metastatic sites [3]. SBRT does not replace systemic therapy but rather can augment its effects on focal areas of gross disease, as well as metastatic lymph nodes.

Previous published experiences in our Institute have shown how SBRT for abdominal targets resulted to be feasible with good early local control rate and acute toxicity profile [4]. In particular, the medium-term clinical outcome of hypofractionated SBRT seemed to be promising in a series of patients with either a solitary metastasis or oligometastases from different tumors to abdominal lymph nodes [5].

Recently, two new technological platforms have been made available to clinical practice in radiation therapy departments. Firstly, Volumetric Modulated Arc Therapy (VMAT) in its RapidArc® format, allowed a significantly time reduction to deliver complex intensity modulated plans, permitting to treat with hypofractionated regimes within few minutes [6-8]). Secondly, there has been increasing interest into the clinical use of linear accelerators (LINAC) with photon beams generated without usage of the flattening filter [9-15]. It seems possible to predict a reduction of out-of-field dose when flattening filter free (FFF) beams are used. This is mainly related to the reduced head scattering and the residual electron contamination. FFF beams should therefore lead to reduced peripheral doses and patients can benefit by decreased exposure of healthy tissue to scattered doses outside the radiation field. Removal of the flattening filter implies also the possibility to deliver treatments with higher dose rates, up to factor 4 at 10 MV, and with a much higher dose per pulse. This, beside further improving time efficiency for delivery, might have subsequent potential radiobiology implications; now still unclear and deserving dedicated investigations. While research in the physics domain for FFF beams is increasing, there are few clinical data where FFF beams are applied in clinical practice, particularly in SBRT treatments [16].

Here we reported dosimetric and early clinical data of SBRT with FFF in isolated lymph node oligometastatic patients.

## Methods

### Patient population

Between October 2010 and March 2012, a total of 34 patients were treated with SBRT for isolated lymph node metastases in abdomen and or pelvis on a Varian TrueBeam^TM^ treatment machine (Varian Medical Systems, Palo Alto, CA, USA) using RapidArc technology. Twenty-eight of those patients were treated with SBRT and FFF beams; among them 3 were lost to follow-up. The following will refer to this 25 patient cohort with follow-up, for a total of 28 treatments. Patient data were collected and retrospectively analyzed. Table 1 summarized inclusion criteria and Table 2 summarized demographic data of the population of study. Patients were stratified for metastatic disease site (abdominal or pelvic).

**Table 1:** Inclusion criteria

**Age:**	**≥18years**
WHO performance status:	≤ 2
Histologically-proven of primary cancer disease	
M1 stage with primary cancer site radically treated with complete response/resection or stable.	
No other site of disease in progression (a maximum of 3 lymph node sites of disease to treat)	
Diameter:	<5 cm
Abdomen/pelvic site	
No previous surgery or RT in the region to treat	
Informed consent.	

**Table 2:** Demographic patient and treatment data

Patient	Gender (Nb of patients)	
	Male	19
	Female	6
	Age (y)	
	Median (range)	70 (32, 83)
Tumour	Primary (Nb of patients)	
	Colon	6
	Stomach	2
	Biliary tract / pancreas	3
	Breast	1
	Lung	4
	Sarcoma	1
	Ovary	2
	Kidney	3
	Prostate	3
	Metastasis site (Nb of treatments)	
	Abdominal	8
	Pelvic	20
	Nb of metastases (Nb of treatments)	
	Solitary	23
	Oligometastases	5
	CTV volume (cm^3^)	
	Mean±SD (range)	17.4±21.0 (1.2, 103,8)
	PTV volume (cm^3^)	
	Mean±SD (range)	56.8±42.0 (9.6, 185.9)
	Previous chemotherapy (Nb of patients)	
	Yes	20
	No	5

### Dose prescription, simulation procedures and target delineation

Prescription doses were 45 Gy in 6 consecutive fractions of 7.5 Gy for all 28 treatments. The inclusion criteria were: age ≥18years, WHO performance status ≤ 2, histologically-proven of primary cancer disease, M1 stage with primary cancer site radically treated with complete response/resection or stable, no other site of disease in progression (a maximum of 3 lymph node sites of disease to treat), diameter of lymph node Target less than 5 cm, Abdomen/pelvic site, no previous surgery or RT in the region to treat, obtained informed consent.

Chemotherapy, when prescribed, was interrupted from 20 days before the simulation to the first evaluation after the end of SBRT treatment, as scheduled.

Patient preparation for planning CT and each treatment session foresees a 3-hour fast to avoid large displacement of stomach and bowel during daily treatment with respect to planning CT anatomy.

CT scans for planning were acquired for all patients in supine position, with the arms above their head and immobilized with a thermoplastic body mask including a styrofoam block for abdominal compression to minimize internal organ motion. Contrast free and contrast-enhanced CT scans were acquired in free breathing mode at 3 mm slice thickness in the same patient treatment position during the same acquisition session. The abdominal compression was assessed in our clinic to adequately minimize the internal motion, with 4DCT acquisitions in some past cases to assess the residual movement being of < 5mm. Such an immobilization is the standard in our clinic for all stereotactic (or hypofractionated) abdominal irradiations, without adding a 4DCT acquisition for ITV (internal target volume) delineation.

The clinical target volume (CTV) included macroscopic (for practical reasons, the gross tumor volume GTV was not explicitely outlined) and microscopic disease, based on CT as well as on PET imaging when available. Set-up margin was minimised using the cone-beam CT (CBCT) verification before each treatment session. The overall CTV to PTV (planning target volume) margin was of 6–10 mm in all directions, based on previous study on 4DCT acquisitions as mentioned above. Margins were differentiated depending on the lesion location. Since the residual internal organ motion was limited due to abdominal compression, no planning organ at risk volumes (PRV) was defined for any organ at risk (OAR) nor included in the optimization process.

### Planning objectives

Prescription dose was defined as the mean dose to the PTV, aiming to cover the PTV with 95% of the prescribed dose. If organs at risk tolerance doses did not allow that coverage, the prescription was then applied to the mean dose to the CTV, aiming to cover the CTV with 95% of the prescribed dose, and the PTV with 80% of the same prescribed dose. Maximum dose was to be kept below 107% of the prescription.

Main OAR considered were: stomach, duodenum, small bowel, liver, spinal cord, kidneys. Plans were required to meet the following physical dose objectives (which account for the used fractionation):

Stomach and duodenum: V_36G_<1cm^3^

Small bowel: V_36Gy_<3cm^3^

Liver: V_21Gy_<(total liver volume – 700cm^3^)

Spinal cord: D_1cm3_<18Gy

Kidneys: V_15Gy_<35%

### SBRT planning and delivery procedure

Flattening filter-free (FFF) photon beams of nominal 6 or 10 MV from a Varian TrueBeam were used for all 28 treatments, using the maximum available dose rate of 1400 or 2400 MU/min (for 6 or 10 MV FFF, respectively). Plans were individually set-up with the VMAT using the RapidArc® technology. Full arcs were used in 9 cases, while for the other plans partial arcs setting was chosen. Single arc was planned for only 4 cases, and multiple arcs (mostly two) were preferred according to patient anatomy and mutual PTV and OAR location in order to obtain the best adherence to planning objectives for each patient. All dose distributions were computed with the Anisotropic Analytical Algorithm (AAA, version 8.9) implemented in the Eclipse treatment planning system (Varian). The ‘jaw tracking’ option available for TrueBeam facilities was applied during the optimization phase. With this tool the main jaws are driven by the control system to follow the actual minimum MLC aperture during the arc delivery. For multiple lesions the attempt was to keep one single isocentre, but the choice of single or multiple isocentre was driven by the obtained dose distribution in the two competitor plans. Only one case of multiple lesions was treated with two isocentres.

Treatment was possibly delivered in 6 consecutive working days. Treatment delivery was preceded by CBCT image guidance with, whenever needed, on-line couch adjustment. Image matching was performed on bony structures and, when visible, on tumors and/or other soft tissue structures (e.g. main blood vessels).

Prior to the first session, pre-treatment quality assurance was performed using the MatriXX (IBA Dosimetry, Shwarzenbruck, Germany) 2D array of ion chambers (distance between detectors: 0.76 cm) placed in a PMMA slab phantom. The dose at the detectors plane was calculated with the same patient plan in the phantom and compared with measurement. Evaluation was based on γ index, with criteria of 3 mm and 3% as distance to agreement and dose difference. Acceptability was set to 95% of the points passing the threshold of γ<1.

Efficiency of the treatment was evaluated in terms of beam on time, that is reduced by the usage of FFF beams, allowing dose rates up to 2400 MU/min.

### Evaluation of dosimetric data

The quantitative evaluation of plans was performed by means of dose–volume histograms (DVH) data. For PTV and CTV, the values of D_99%_ and D_1%_ (dose received by 99%, and 1% of the volume) were defined as metrics for minimum and maximum doses; V_95%_ (the volume receiving at least 95% of the prescribed dose), homogeneity as Standard Deviation parameter of DVH and D_5%_-D_95%_, as well as the conformity index CI (defined as the ratio between the patient volume receiving 95% of the prescription dose and the PTV volume) were also reported.

For OARs, the analysis included the mean dose, the maximum dose expressed as D_1%_, and a set of appropriate V_X(Gy)_ and D_Y(% or ccm)_ values.

Average cumulative DVH for PTV, CTV and OARs were determined from the individual DVHs. These histograms were obtained by averaging the corresponding volumes over the whole patient cohort for each dose bin of 0.05 Gy.

### Evaluation of clinical data

Patients had clinical evaluations planned before and during the treatment, at the end of the last fraction. Then, the first follow-up visit was scheduled, based on site and general condition, within 45–120 days from the end of the treatment, then every 3–4 months, Acute toxicity induced from the radiation treatment were scored and recorded according to the National Cancer Institute’s Common Terminology Criteria for Adverse Events (CTCAE version 3.0).

Local control was evaluated on CT images at the first follow-up for all cases; patients who had a PET scan before SBRT, the same metabolic examination and response were also evaluated during the follow-up and scored according PERCIST criteria. Local control evaluation was scored according to the WHO criteria as complete remission (CR), partial remission (PR), stable disease (SD) for each treatment. Distant progression disease (PD) scored the clinical results on possible lesions out of the local target.

Although obviously very early, a first assessment of initial treatment outcome was performed at first and second follow up visits.

## Results

### Dosimetric data

**Figure 1:**
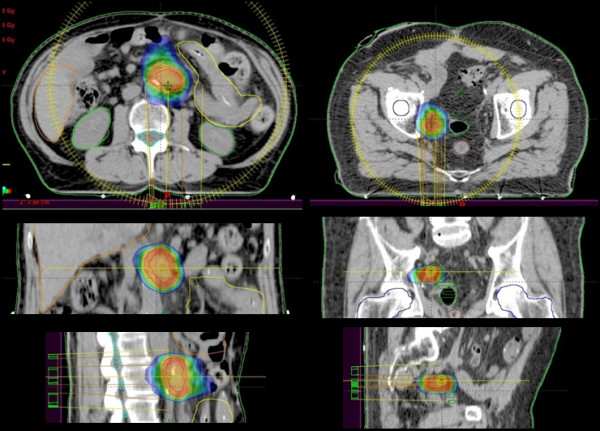
Dose colorwash from 50% dose level for an abdominal lymph node case (left) and a pelvic lymph node case (right).

**Figure 2:**
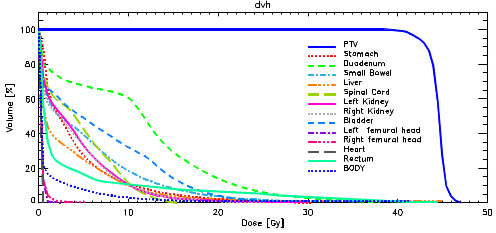
Average DVH of the main structures on the whole patient cohort.

Figure 1 illustrates two examples of dose distributions of abdominal and pelvic lymph node treatments in axial, sagittal and coronal views. Figure 2 presents the average cumulative dose volume histograms for the PTV and the involved organs at risk. Table 3 shows results from DVH analysis for the analyzed structures.

**Table 3:** Dose distribution statistics

**Structure**	**Parameter**	**Objective**	**Mean±SD**	
				**From Bignardi**
**PTV**	Mean [Gy]	50.0	44.9±0.7	44.5±0.3
Volume [cm^3^]	D_5%-95%_ [Gy]		2.8±1.5	3.8±0.8
56.7±42.0	Standard Dev [Gy]		0.9±0.5	
	D_1%_ [Gy]	<48.15	46.2±0.8	46.4±0.3
	D_99%_ [Gy]	>42.75 (36)	42.0±2.4	40.8±1.0
	V_95%_ [%]		97.7±3.2	90.2±5.2
	CI_95%_	1.0	1.1±0.1	1.0±0.1
**CTV**	Mean [Gy]		45.1±0.8	45.0±0.0
Volume [cm^3^]	D_5%-95%_ [Gy]		1.4±0.8	1.4±0.3
17.4±21.0	Standard Dev [Gy]		0.4±0.3	
	D_1%_ [Gy]	<48.15	45.9±0.8	46.0±0.2
	D_99%_ [Gy]	>42.75	43.8±1.8	44.1±0.2
	V_95%_ [%]	100	99.7±1.3	100.0±0.0
**Stomach**	Mean [Gy]		4.2±1.9	4.0±5.5
Volume [cm^3^]	D_1%_ [Gy]		17.9±9.0	12.2±11.7
228.8±129.2	V_10Gy_ [%]		11.2±12.1	
	V_15Gy_ [%]		5.0±6.2	
	V_36Gy_ [cm^3^]	<1	0.05±0.08	0.1±0.5
**Duodenum**	Mean [Gy]		11.0±7.5	7.0±5.6
Volume [cm^3^]	D_1%_ [Gy]		22.9±14.2	22.4±13.8
34.5±24.2	V_10Gy_ [%]		60.8±42.6	
	V_20Gy_ [%]		14.4±19.2	
	V_36Gy_ [cm^3^]	<1	0.09±0.19	0.5±0.9
**Small Bowel**	Mean [Gy]		5.1±3.5	3.0±2.3
Volume [cm^3^]	D_1%_ [Gy]		23.3±6.7	18.0±10.8
1900.9±1764.5	V_10Gy_ [%]		18.6±17.0	
	V_20Gy_ [%]		3.6±4.6	
	V_36Gy_ [cm^3^]	<3	2.9±3.9	0.1±0.2
**Liver**	Mean [Gy]		3.4±2.5	3.6±3.9
Volume [cm^3^]	D_1%_ [Gy]		19.2±14.5	20.2±17.3
1450.5±287.3	V_10Gy_ [%]		10.2±9.6	
	V_20Gy_ [%]		2.0±2.6	
	(V_liver_-700)-V_21Gy_ [cm^3^]	>0	724±285	
**Spinal Cord**	D_1%_ [Gy]		10.1±3.2	7.8±2.3
Volume [cm^3^]	D_1ccm_ [Gy]	<18Gy	9.5±3.1	
32.6±19.7	V_10Gy_ [%]		12.2±19.9	
**Left Kidney**	Mean [Gy]		4.2±3.4	2.2±2.5
Volume [cm^3^]	D_1%_ [Gy]		11.6±8.7	8.3±8.1
162.4±36.8	V_15Gy_ [%]	<35	3.1±7.5	1.7±5.9
**Right Kidney**	Mean [Gy]		3.9±3.6	3.1±2.9
Volume [cm^3^]	D_1%_ [Gy]		10.7±6.9	10.4±11.2
163.4±39.9	V_15Gy_ [%]	<35	2.9±8.6	2.8±7.1

We compared the present data using FFF beams with previous dosimetric data of the same group, for the same type of stereotactic abdominal lymph node metastases treatments and fractionation with RapidArc® technology [8]. All dosimetric results are in line with what we previously published without FFF, as reported in the last column of Table 3 for the published data. The differences between the two dataset are that they are based on a different group of patients, and that the published data refer to standard flattened beams, while the present work reports on FFF results.

In particular a very highly degree of conformality is available in the present study with FFF, with very high volume fraction receiving the 95% of the dose prescription: around 98 and 100% of the PTV and CTV respectively. The PTV coverage was 90% for non-FFF beams.

For OARs, it was possible to respect the planning objectives in all the cases, also the V_36Gy_ for stomach, duodenum and bowels. Dose reduction in the CTV to PTV margin (requiring minimum dose to PTV higher than 80% while keeping the 95% coverage for CTV) was required for organs at risk tolerance dose in three cases.

Delivery accuracy determined by the pre-treatment quality assurance was recorded, for the patients of this study, as the percentage of the point passing the gamma criteria of 3 mm/3%. The average of this value over all the patient cohort was 99.2±0.5, with a range of 97.3-99.9. All cases were carefully analysed, not only fixing the attention on the number of gamma passing point, in the whole dose map, and considered acceptable.

Arc delivery with FFF beams was with a maximum of 2400 MU/min for 10 FFF beam, with an average beam on time of 1.47±0.44 min [range: 0.82-2.02 min]. For a comparison, the plans were delivered also with a maximum dose rate of 600 MU/min, that is the usual maximum dose rate available in normal linacs using standard flattened beams. With this setting, the average beam on time would have been 3.49±0.81 min [range: 2.52-5.72 min] and thus, for this dose per fraction, the maximum dose rate of 2400 MU/min reduced the beam on time more than 200%.

### Clinical results

Clinical results as acute gastrointestinal GI toxicity as well treatment outcome are summarized in numbers in Table 4.

**Table 4:** Toxicity and response results

G/I toxicity	Nb of patients (total 25)	
	Grade 0	21
	Grade 1	1
	Grade 2	3
	Grade 3-4	0
Response	Nb of treatments (total 28)	
	At first Follow-up:	
	CR	11
	PR	13
	SD	4
	Nb of treatments (total 28)	
	At median Follow-up of 152 months:	
	CR	13
	PR	9
	SD	6

All 25 FFF SBRT patients completed the treatment, as programmed, with no interruptions.

Acute gastrointestinal toxicity was minimal: one patient showed Grade 1 gastrointestinal toxicity, as gastralgia. Three other patients presented Grade 2 toxicity, one with gastric pyrosis, one with epigastralgia and one with nausea/vomiting. No severe acute toxicity with Grade 3 or more was recorded. All toxicities were recovered within one week (G1 without intervention, G2 with symptomatic drugs).

No late toxicity, as per the rather short follow-up time, was found.

The median follow-up was 195 days (range 48–589 days). All patients had at least the first follow up at a median of 92 days (minimum 47 days) from the end of the radiation treatment.

At the first follow-up, early clinical outcome was assessable at diagnostic evaluation of last control with PET and/or CT in 25 patients (28 treatments). At the end of the first follow-up evaluation, a complete response (according to World Health Organization criteria) was found in 11 cases. A partial response was achieved in 13 cases. The overall response rate was of 86% (24/28 treatments). Four patients had stable disease. No local progression was found.

The results at the median follow up of 195 days (18 patients had a second follow-up) are: complete response in 12 cases, partial response in 11, stable disease in 5, with an overall response rate of 82%; no local progression was recorded.

Of the patient cohort of this study, 6 presented distant progressive disease (outside the treated region) at the first follow-up. This number increase to 15 for the 195 median days follow-up.

## Discussion and conclusions

Rationale of local therapy for oligomestases is that when primary cancer is controlled, the solitary or few metastases can be cured locally to effort systemic therapy [1,2]. Actually, few published data do exist on local control rates of radiotherapy in the context of isolated or few lymph node metastases. Although dose and fractionation schedules are extremely heterogeneous, early data from some recent series are promising in term of local control rates [3]. Because small volumes are irradiated for metastatic lymph nodes, a dose escalation might improve efficacy without prohibitive toxicity.

Most of reported experiences regarded oligometastatic lymph node in pelvis or abdomen and are summarized in Table 5.

**Table 5:** Clinical details of published studies of SBRT for oligometastases lymph nodes

**Reference**	**Primary**	**N° of patients**	**Radiation Dose**	**Median FUP [months]**	**Outcomes**
Jereckzek et al. [17]	prostate	34	30 Gy in 4/5 fractions	16.9	LC: 91% at 17 months
Choi et al. [18]	cervix	30	33-45 Gy in 3 fractions	15	LC:67.4% at 4 years
Kim et al. [19,20]	gastric	7	36/51 in 3/fractions	26	OS:43% at 3 years
Bignardi et al. [5]	miscellaneous	19	45 in 6 fractions	12	LC: 77,8% at 1 year

*LC*= local control; *OS*= overall survival.

The results of the current study with SBRT by RapidArc® and FFF, at the median follow up of 195 days confirm a response rate of 82% and no local progression was recorded. Local control provided by the current initial experience may be potentially significant for preserving quality of life and delaying further systemic treatments. Obviously, the most significant criticism remain: a) the heterogeneity of the population of study, composed by a miscellaneous of cases from different primary tumors (see Table 1) b) the short follow-up (median of 195 days), c) the retrospective nature of the study in patient data analysis. Nevertheless, the end point of the study was to define the dosimetric and early clinical results of SBRT by RapidArc® with FFF, and this finding was largely confirmed: all plan objectives were met and no toxicity were recorded in acute setting, achieving an intial high rate of local control.

Concerning FFF beams, previous our report showed as the treatment delivery with FFF beams for abdominal lesion is faster and more efficient, improving patient’s comfort and thus reducing intra-fraction motion, characteristic that becomes particularly important in SBRT [21]. Some preliminary studies for SBRT using FFF beams are present in literature [11,22] and in a recent study performed in our institute it was described our early experience in the use of FFF beams for SBRT treatments including liver metastases, lung primitive and metastases, isolated abdominal lymph nodes, adrenal glands, and pancreas [16]. Also in the present report, acute toxicity profile was excellent: no severe acute Grade 3 toxicity or more was recorded and all toxicities (2 cases of Grade 1 and 3 cases of Grade 2) were recovered within one week.

From the radiobiological point, some literature starts to show that FFF beams, relatively to standard flattened beams, have higher efficacy, possibly in reducing survival fraction for the same delivered physical dose, due to the higher dose per pulse of such beams (up to 4 times) [23]. This fact is today only a suggestion coming from sparse laboratory studies on cell lines, and not yet clinically proven in terms of patient studies. In principle the improvement is present, but the increased cell killing effect is rather minimal, so difficult to be proven at the present stage with small clinical studies. To clinically demonstrate the differences in cell killing effect between standard beams and beams with high dose per pulse, clinical trials should be set on a rather large scale. Also the time factor, not only the dose per pulse, is known to have a role the on radiation induced damage. This was subject of different studies where, both on theoretical bases and in vitro irradiations [24,25] it is demonstrated that the shorter is the treatment session time, the higher is the tumor control probability for the same physical delivered dose. Although not yet clinically proven, all those studies suggest the possible benefit in using FFF beams, for which the present study can present a practical feasibility.

From the technical viewpoint, a first point to underline about such beams is the known reduction of peripheral dose, coming from the absence of the flattening filter that reduces the scattered dose from the linac head. To enhance this effect, with the TrueBeam linac it is also possible to allow the jaws to follow the MLC movement, minimizing the field area shielded by only the MLC: this option further reduces the dose in the proximity of the target, potentially improving the dose fall-off toward the critical structures and surrounding tissues. Technically speaking, no specific study has been undertaken up to now to systematically quantify the peripheral dose reduction when using the jaw tracking option, as its application is part of the optimization process, ending in different MLC sequencing if the option is used or not. As a second technical point to mention is the use of the abdominal compressor to minimize the internal organ motion. This was considered adequate in our institution, but the possible use of 4DCT acquisition for ITV delineation, in conjunction to the compressor, could be a more precise solution to better include the motion management, an essential point for SBRT in this anatomical region. The 4DCT is indeed going to be included in our next clinical studies for thoracic and abdominal stereotactic irradiations.

Considering these promising results, a prospective study of dose escalation in 4 fraction of SBRT with FFF beams for pelvic/abdominal lymph node oligometastatic patients (genito-urinary and gynecology primary) was recently proposed and accepted by internal ethical committee of our Institute and preliminary results will be reported in a further study.

## Competing interests

L. Cozzi acts as Scientific Advisor to Varian Medical Systems and is Head of Research and Technological Development at the Oncology Institute of Southern Switzerland, Bellinzona. Other authors report no conflict of interest.

## Authors’ contributions

FA, MS and AF coordinated the entire study. Data collection and clinical data analysis were conducted by FA, EC, PN, AT, TC, AMA, MS. Dosimetric data collection and analysis were conducted by AF, AC, FL, GR, LC, PM, ST. All authors read and approved the final manuscript.
